# Potential mechanisms for osteopathic manipulative treatment to alleviate migraine-like pain in female rats

**DOI:** 10.3389/fpain.2024.1280589

**Published:** 2024-02-05

**Authors:** Katherine Byrd, Makayla Lund, Yan Pan, Brandon H. Chung, Kaitlyn Child, Danny Fowler, Jared Burns-Martin, Mythili Sanikommu, Hallie Henderson, Caroline Gregory, Regina K. Fleming, Jennifer Yanhua Xie

**Affiliations:** ^1^ Department of Biomedical and Anatomical Sciences, New York Institute of Technology College of Osteopathic Medicine at Arkansas State University, Jonesboro, AR, United States; ^2^ Department of Osteopathic Manipulative Medicine, New York Institute of Technology College of Osteopathic Medicine at Arkansas State University, Jonesboro, AR, United States

**Keywords:** osteopathic manipulative treatment, migraine-like headache, allodynia, trigeminal nucleus caudalis, trigeminal ganglia, calcitonin gene-related peptide

## Abstract

**Introduction:**

Migraines are the leading cause of disability in the United States, and the use of non-pharmaceutical treatments like osteopathic manipulative treatment (OMT) has shown promise. Despite its potential, the lack of mechanistic understanding has hindered widespread adoption. This study aims to investigate the efficacy of OMT in treating acute migraines and unravel its underlying mechanisms of action.

**Methods:**

Female rats were subjected to a “two-hit” approach to induce migraine-like pain. This involved bilateral injections of Complete Freund's Adjuvant (CFA) into the trapezius muscle (1st hit) followed by exposure to Umbellulone, a human migraine trigger, on Day 6 post-CFA (2nd hit). Soft tissue and articulatory techniques were applied to the cervical region for acute abortive or repeated prophylactic treatment. Cutaneous allodynia and trigeminal system activation were assessed through behavioral tests and immunohistochemical staining.

**Results:**

Following Umbellulone inhalation, CFA-primed rats exhibited periorbital and hind paw allodynia. Immediate application of OMT after Umbellulone inhalation as an abortive treatment partially alleviated cutaneous allodynia. With OMT applied thrice as a prophylactic measure, complete suppression of tactile hypersensitivity was observed. Prophylactic OMT also prevented the increase of c-fos signals in the trigeminal nucleus caudalis and the elevation of calcitonin gene-related peptide expression in trigeminal ganglia induced by CFA and Umbellulone exposure at 2 h post-inhalation.

**Discussion:**

These findings provide mechanistic insights into OMT's migraine-relief potential and underscore its viability as a non-pharmacological avenue for managing migraines.

## Introduction

Migraine, characterized by recurrent unilateral throbbing cephalic pain along with hypersensitivity to external stimuli, poses a significant debilitating challenge (1, 2). While the precise triggers for migraine attacks remain elusive, the activation of the trigeminovascular system stands as a pivotal factor in pain development (1, 3). Trigeminal activation has been recognized as a predictive indicator for migraine onset among sufferers (4). This activation leads to the release of excitatory neurotransmitters, especially calcitonin gene-related peptide (CGRP) from dural afferent terminals (5). Substantial evidence now supports a crucial role of CGRP in the pathophysiology of migraine (6, 7). Release of CGRP from nociceptors innervating the cranial meninges is thought to contribute to neurogenic vasodilation and to promote sensitization and activation of these fibers (7, 8). Sustained activation of peripheral nociceptors can elicit sensitization of the second-order neurons of the trigeminal *nucleus caudalis* (TNC) (3, 4) resulting in enhanced nociceptive inputs to higher brain centers including the thalamus, hypothalamus and cortical sites, collectively manifesting as migraine pain (4, 9).

Current migraine treatments, encompassing triptans, nonsteroidal anti-inflammatory drugs (NSAIDs) and CGRP antibodies/antagonists, yield response rates of approximately 50% (10–15). Due to limited efficacy or adverse effects, many migraine patients seek complementary approaches such as nutrition/diet adjustments, exercise, traditional Chinese medicine, and manual treatments like osteopathic manipulative treatment (OMT) (16–20). These options are cost-effective, well-tolerated, and generally entail minimal side effects. Nevertheless, the substantiation of their effectiveness, determined through double-blinded clinical trials, remains generally insufficient.

OMT is a form of manual treatment that targets the musculoskeletal system and emphasizes the intricate interplay between bodily structure and function. Involving soft tissue mobilization, joint manipulation, myofascial release, and other approaches, OMT aims to enhance blood circulation, diminish muscle tension, and foster the body's innate self-healing mechanisms. Although some reports suggest that OMT effectively reduces the frequency, intensity, and duration of migraine headaches and improve patients' quality of life (16, 19–21), the evidence supporting its efficacy for migraines remains inconclusive or of limited quality (20). Elucidating the potential mechanisms underpinning OMT's effects would substantially bolster the acceptance of this treatment strategy and promote evidence-based medical practice.

While migraines commonly associate with the ophthalmic division (V1) of the trigeminal nerve, over two-thirds of migraine patients also experience neck pain during migraine episodes or in the prodrome/postdrome phases, aligned with the receptive field of the greater occipital nerve (GON) originating from the C2 spinal nerve root (22–26). It has been suggested that some of the trigeminal afferents terminate at the C2 spinal segment (27–29). The anatomical and functional convergence of trigeminal and upper cervical afferent input at the trigeminal nucleus caudalis (TNC) might contribute to the concurrent head and neck pain (30). Furthermore, the functional convergence of sensorimotor fibers in the spinal accessory nerve (CN XI) and upper cervical nerve roots, along with the descending tract of the trigeminal nerve, may underlie the referral of cervical pain to the head (31). Nociceptive inputs from cervical muscles like the splenius capitis, upper trapezius, and sternocleidomastoid can sensitize the TNC, potentially intensifying cephalic pain (23, 30, 32). Massaging over the GON has been shown to reduce the intensity of migraine pain (33).

In this study, we developed a new migraine model built upon the established models characterized by the Durham (34) and Porreca (35) groups. This “two hit” model entails priming animals with an immunogenic inflammatory agent, Complete Freund Adjuvant (CFA), injected bilaterally into the trapezius muscles at a low dose to induce mild cervical inflammation and establish a state of “latent sensitization” (1st hit). By Day 5 post-CFA injection, the animals display normal sensory thresholds at the periorbital region. However, upon exposure to umbellulone (2nd hit), a major volatile molecule emitted by the “heacache tree” Umbellularia californica, cutaneous allodynia and activation of the TNC developed. Umbellulone, identified as a transient receptor potential ankyrin type 1 (TRPA1) agonist, interacts with this ion channel highly permeable to sodium and calcium ions (36, 37). TRPA1 receptors are primarily expressed in slowly conducting primary afferent neurons (38, 39), which mediate nociceptive and cold receptive functions (40). Activation of TRPA1 sensitizes meningeal afferents, potentially facilitating headache generation (41, 42).

Utilizing this new migraine model, we investigated the effects of OMT on migraine-like pain behavior and pathophysiology. Drawing from empirically employed human soft tissue (43) and articulatory (44) techniques by osteopathic practitioners, we adapted an OMT protocol for rats. This approach involved direct passive manipulation along the neck and upper torso regions to address migraines and cervicogenic headaches. We administered OMT to the neck muscles and cervical vertebra of the rats before or after the migraine trigger and assessed the efficacy of acute or preventative OMT in treating migraines in this model. Additionally, we explored TNC activation and CGRP expression in trigeminal ganglia (TG) tissues, aiming to glean mechanistic insights into how OMT relieves migraines.

## Methods

### Animals

Adult female Sprague-Dawley rats with an initial weight of 175–200 g (Charles River) were used for the experiments. The rats were housed in groups of 4 per cage on a 12 h light-dark cycle (lights on at 8 AM). They have *ad libitum* access to standard autoclaved lab chow and water. The allocation of animals to different treatment groups was randomized ensuring impartial distribution. All experiments were performed by experimenters blinded to the treatment groups and the blinding codes were not revealed until after the data analysis was completed. Group size for each experiment was determined by power analysis (see the Statistical Analysis below).

### Rat migraine model

The rats were anesthetized with 2% isoflurane at 1 L/min. Trigeminal sensitization was induced by injecting CFA (Sigma-Aldrich, St. Louis, MO) diluted 1:1 in 0.9% sterile saline. The injections were distributed across ten different sites in the trapezius muscle between C1 and C4 vertebrae. Each side received 5 injections of 10 μl each, resulting in a bilateral total of 100 μl (50 μl per side). After the injections, the animals were allowed to recover in their home cages for 6 days. On the 6th day, rats were placed in a Plexiglas chamber (dimensions: 23 × 9.5 × 9 cm height) connected to an O_2_ tank for exposure to umbellulone (Sigma-Aldrich, St. Louis, MO). Umbellulone was dissolved completely in DMSO and then diluted 20-fold with deionized water (DiH_2_O) to achieve a final concentration of 0.05 M immediately before use. A small cotton ball soaked with 50 μl of umbellulone was placed within a small tube positioned at the chamber entrance, allowing continuous delivery of umbellulone to the chamber at a flow rate of 2 L/min for 30 min. Following the umbellulone exposure, rats were returned to their home cages. The sham treatment group received a vehicle solution comprising 5% DMSO and 95% DiH_2_O for 30 min using the same delivery method.

### Behavioral testing for tactile hypersensitivity

**Figure 1:**
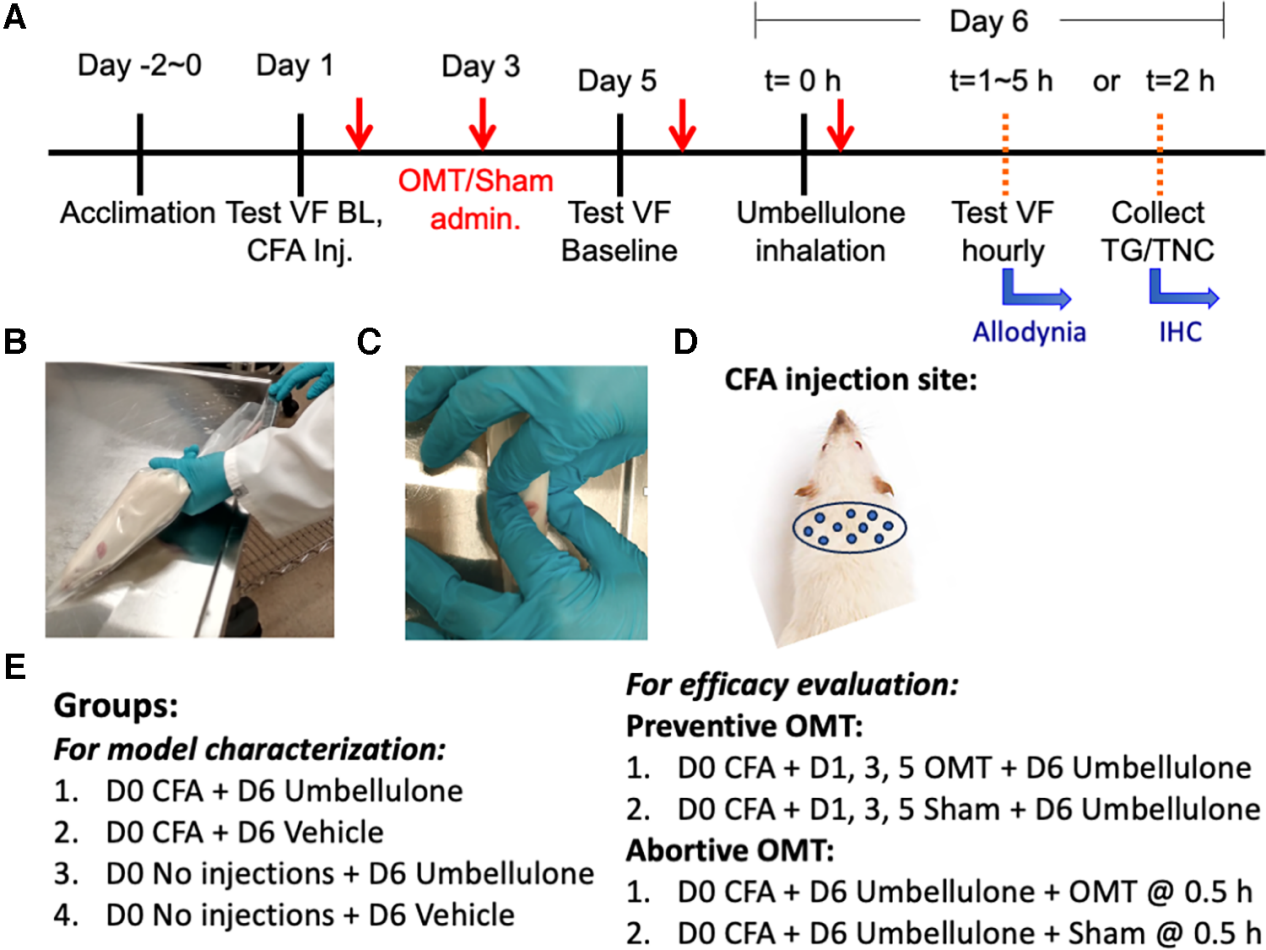
Experimental plan and application of OMT. Panel (**A**) illustrates the study design and experimental timeline. The animals were gently restrained inside the plastic decapicones while awake (**B**) OMT were applied to the neck region using 1 min soft tissue techniques followed by 1 min of articulatory techniques, as depicted in Panel (**C**) Sham animals were only restrained for 2 min without any manipulations. The injection sites for CFA are illustrated in Panel (**D**) the experimental groups are listed in Panel (**E**).

All behavioral evaluations were carried out between 9 AM and 3 PM (35). Prior to testing, rats were acclimated to the Plexiglas testing chambers (33 × 17 × 20 cm height) containing a wired mesh floor over a period of 1 h daily for 5 consecutive days. Mechanical thresholds were determined in response to a series of calibrated von Frey (VF) filaments (Stoelting, Wood Dale, IL) applied in increasing force to the cutaneous tissue. The cutoff threshold was set at 8 g for the periorbital region and 15 g for the hind paw region. Baseline thresholds were determined prior to any experimental procedures. A positive response, indicated by head or paw withdrawal upon filament contact, was recorded. The up-down method was adopted, and withdrawal thresholds were calculated using the Dixon method (45). The experimental procedure is illustrated in Figure 1.

### Osteopathic manipulative treatment (OMT) procedure

Adapting human manipulations, we introduced a modified soft tissue technique involving bilateral lateral stretch and an articulatory technique applied to cervical vertebrae in rats (Figure 1). Rats were restrained in a decapicone on a treatment table. An experimenter positioned a finger on each side of the rat's neck. Traction was applied bilaterally to the trapezius muscle, moving up and down along the neck for 1 min to relax local tissues. Subsequently, the articulatory technique was applied by gently translating cervical vertebrae side-to-side to remove any restrictive barriers by guiding the joint through its full range of motion for an additional minute, focusing on the neck. Following these maneuvers, traction was released, and palpation ceased. The rat was then returned to its home cage. Dr. Regina Fleming, D.O., a practitioner experienced in similar techniques, developed this treatment regimen. The form and duration of the treatment was determined by Dr. Fleming based on the desired outcomes, primarily enhanced relaxation of the cervical muscles and an augmented translatory motion bilaterally in the cervical spine, assessed through her fingers during the preliminary studies.

### Immunohistochemical staining

Immunostaining was conducted following previously described methods (46). Briefly, at 2 h post-umbellulone inhalation, the animals were deeply anesthetized with ketamine/xylazine (100/12 mg/kg, i.p.) and subjected to transcardial perfusion with 4% paraformaldehyde. TG and TNC tissues were harvested and cryoprotected with 30% sucrose. The tissues were then dissected into thin slices of 15 μm (TG) and 30 μm (TNC) at −20°C using a cryostat. Slides from each experimental condition were washed in phosphate-buffered saline (PBS) 3 times for 3 min each, followed by blocking using a PBS solution containing 0.2% Tween20, 1% bovine serum albumin, and 2% glycine for 2 h at room temperature. Primary antibodies, diluted in PBS containing 3% goat serum and 0.05% Triton X-100, were incubated with the tissues over night at 4°C. Alexa Fluor secondary antibodies, diluted in PBS, were incubated with tissues at room temperature for 1 h. The VECTASHIELD medium was employed for tissue mounting, and fluorescence microscopy was performed using the BioTek Cytation-5 Bioimager/Plate Reader (Winooski, VT, USA). Gen-5 analysis software was utilized to quantify the number of cells expressing the specific markers using an unbiased manner based on fluorescence. To ensure consistent comparison for quantifications, tissues from all groups were processed in parallel to account for batch-to-batch variations. Within each batch, the exposure time, region of interest and quantification threshold were set by an experimenter blinded to the grouping information based on the entire batch of samples. Cell counts were obtained from 3 independent experiments, and 4–6 standardized regions of interest encompassing the TG or TNC were analyzed for each tissue sample. Mean values were determined for each animal, and data were reported as average cell counts per group with individual values indicated by the scatter plots. The detailed information of the antibodies is listed in Table 1.

**Table 1:** Antibodies used for IHC studies.

Antibody	Vendor (Lot No.)	Dilution	Incubation
C-Fos (9F6) rabbit mAb	Cell signaling (2250s)	1:2,000	Overnight at 4°C
CGRP (D5R8F) rabbit mAb	Cell signaling (14959s)	1:400	Overnight at 4°C
Alexa Fluor 594 goat anti-rabbit lgG(H + L)	Thermo Fisher scientific (2506100)	1:1,000	1 h at room temperature

### Statistical analysis

All statistical analyses were performed using GraphPad Prism 9 software. Sample size was determined using G*Power3.1 based on historical data, with a set power (1-*β*) of 0.95 and an *α* error of 0.05. Data were presented as mean ± SEM. One-way ANOVA followed by Tukey's multiple comparison *post hoc* analysis or Two-way ANOVA followed by Dunnett's multiple comparison *post hoc* analysis was employed as appropriate. Statistical significance was set at *P *≤ 0.05.

## Results

### Umbellulone induced tactile allodynia in CFA-primed rats

**Figure 2:**
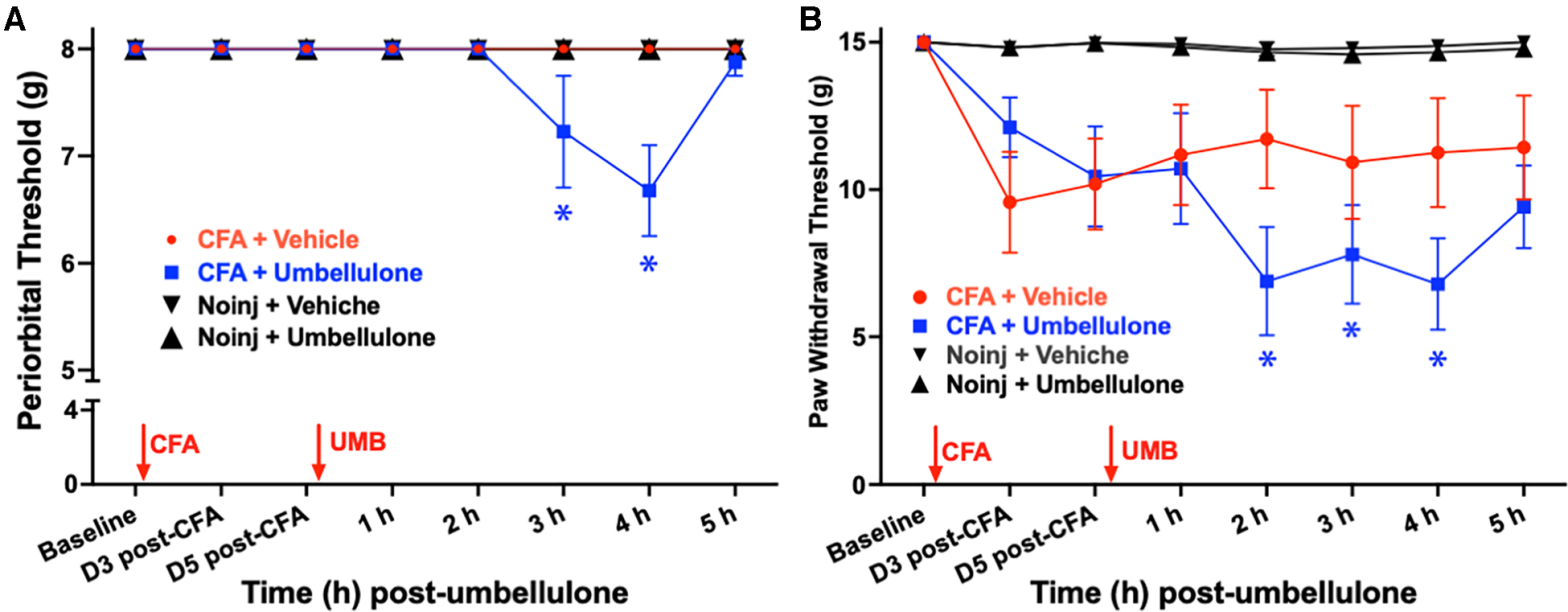
Effects of CFA priming and umbellulone inhalation on periorbial and hind paw tactile thresholds. Rats received bilateral injections of CFA (1:1 diluted with saline, 10 × 10 μl) into the trapezius muscle. Periorbital tactile threshold was measured at baseline (BL), as well as on Days 3 and 5 post-CFA, and hourly for 5 h on Day 6 after inhalation of umbellulone (50 mM, 50 μl)*.* Cephalic allodynia developed at 2 and 3 h post-umbellulone inhalation in CFA-primed animals, while hind paw allodynia was observed at 2, 3 and 4 h post-umbellulone inhalation. Statistical analysis was performed using two-way ANOVA *post hoc* Dunnett's multiple comparison test. *P < 0.05 compared to the BL within the same group. *N* = 8/group.

In the novel rat migraine model induced by umbellulone inhalation in CFA-primed female SD rats (Figure 2), we assessed the tactile hypersensitivity in the periorbital and hind paw regions. Initial baseline periorbital and hind paw withdrawal thresholds were 8.0 ± 0.0 & 15.0 ± 0.0 g, respectively, in naïve rats. CFA (5 injections/side, 10 μl each, i.m.) was injected bilaterally into the upper trapezius muscle under brief isoflurane anesthesia. Tactile threshold was evaluated on Days 3 and 5 post-CFA. Periorbital allodynia was absent at both time points with the tactile threshold remained at 8.0 ± 0.0 g. The hind paw threshold did not decrease significantly, either, compared to the baseline (9.6 ± 1.2 and 10.2 ± 0.9 g at Day 3 and 5 post-CFA, respectively, *N* = 8/group, *P* > 0.05).

On day 6, rats were exposed to umbellulone (50 mM, 50 μl) via inhalation for 30 min in a Plexiglas chamber using 2% O_2_. After umbellulone exposure, rats were placed in the VF chambers, and cutaneous tactile thresholds were assessed hourly for 5 h afterwards. CFA-primed animals exhibited significant periorbital and hind paw allodynia following umbellulone administration. The average periorbital threshold was reduced to 7.2 ± 0.5 and 6.6 ± 0.4 g at 3 & 4 h post-umbellulone, respectively, in the CFA + Umbellulone group (*P* < 0.05 compared to the corresponding baseline thresholds of each group or at the same time point of No Injection + Umbellulone group). In contrast, no change was observed in the CFA + Vehicle or No injection + Umbellulone groups. Similarly, the average hind paw threshold was reduced to 6.9 ± 1.8, 7.8 ± 1.7 and 6.8 ± 1.6 g at 2, 3 & 4 h post-umbellulone, respectively in CFA + Umbellulone group (*P* < 0.05 compared to the corresponding baseline thresholds of each group or at the same time point of No Injection + Umbellulone groups). The animals in the CFA + Vehicle or No Injection + Umbellulone groups did not show significant reduction of the hind paw tactile threshold from the baseline at any of the measured time points. *N* = 8/group.

These findings suggest that mild inflammation induced by CFA in the trapezius muscle created a “latent sensitization” state in naïve rats, rendering them more susceptible to the migraine trigger—umbellulone. It is important to note that the vehicle of umbellulone (5:95 DMSO:H_2_O) did not elicit noticeable effects in CFA-treated rats. Thus, both CFA priming and TRPA1 agonist umbellulone were necessary for inducing cephalic allodynia in naïve rats. This “double-hit” strategy replicates the increased susceptibility to sub-threshold triggers observed in migraineurs, where the control group remains asymptomatic in the absence of migraine triggers.

### OMT acutely abolished the development of cutaneous allodynia

**Figure 3:**
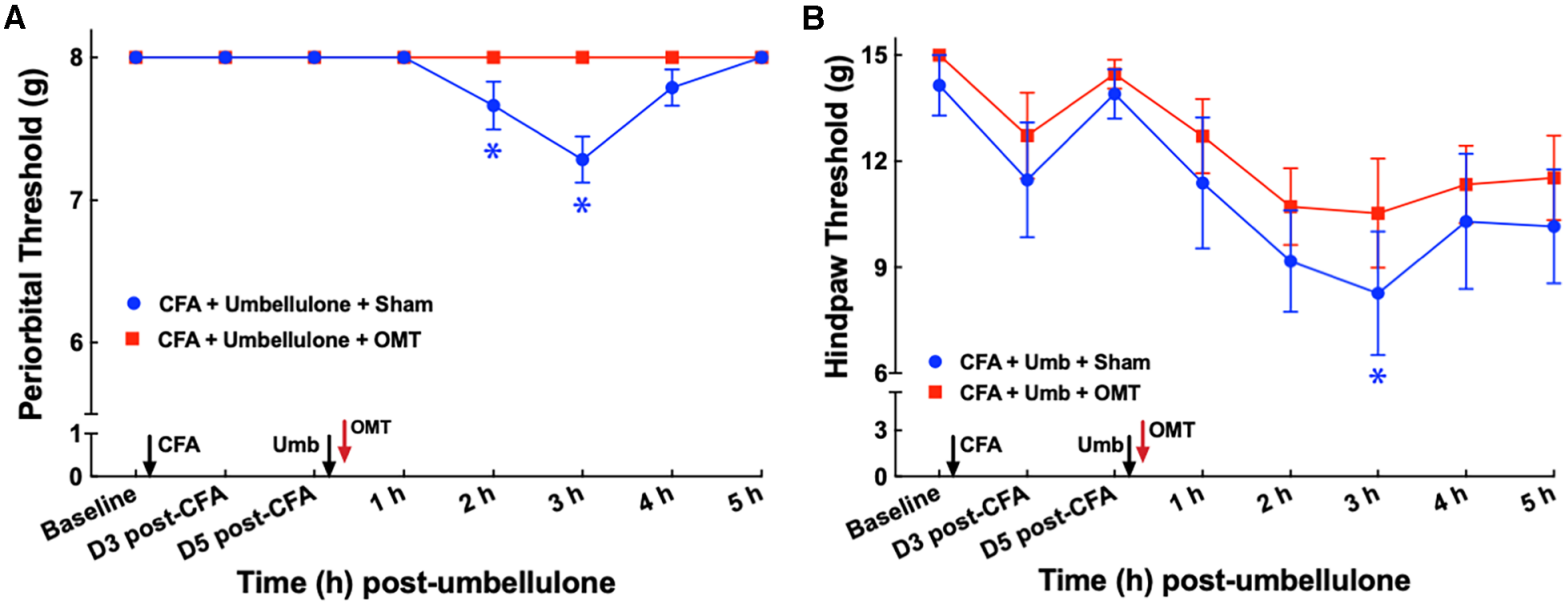
Blockade of periorbital (**A**) and hind paw (**B**) tactile allodynia by OMT as an abortive treatment. Animals were treated with CFA (1:1 dilution with saline) and umbellulone (50 mM, 50 μl) or its vehicle (5% DMSO/95% H_2_O). OMT was applied immediately after umbellulone inhalation in awake animals. Umbellulone significantly decreased the tactile threshold selectively in CFA-primed rats at 2 and 3 h post-dose. OMT inhibited the development of periorbital and hind paw (to a lesser extent) allodynia induced by umbellulone in CFA-primed rats. Statistical analysis was performed using two-way ANOVA *post hoc* Dunnett's multiple comparison test. *P < 0.05 compared to the baseline (BL) within the same group. *N* = 8/group.

After establishing the migraine model, we examined the acute effects of OMT as an abortive treatment for migraine-like pain (Figure 3). Animals received the two hits (CFA and umbellulone) as described above. Immediately after umbellulone inhalation, a single session of OMT (1 min each of soft tissue and articulatory techniques applied to the neck region) or sham (confined in plastic cones for 2 min) was administered. As anticipated, umbellulone administration induced periorbital and hind paw allodynia, shown by the significant reduction of the tactile threshold at 3 h post-sham treatment (7.3 ± 0.2 & 8.3 ± 0.7 g for periorbital and hind paw threshold, respectively; *P* < 0.05 vs. baseline). However, a single application of OMT as an abortive treatment reduced the allodynia in CFA-primed rats. The periorbital tactile threshold was maintained at the baseline level and the hind paw threshold was partially restored to 10.5 ± 0.5 g at 3 h post-Umbellulone and OMT administration (*P* > 0.05 vs. baseline). *N* = 8/group.

### Preventive application of OMT blocked the development of cutaneous allodynia

**Figure 4:**
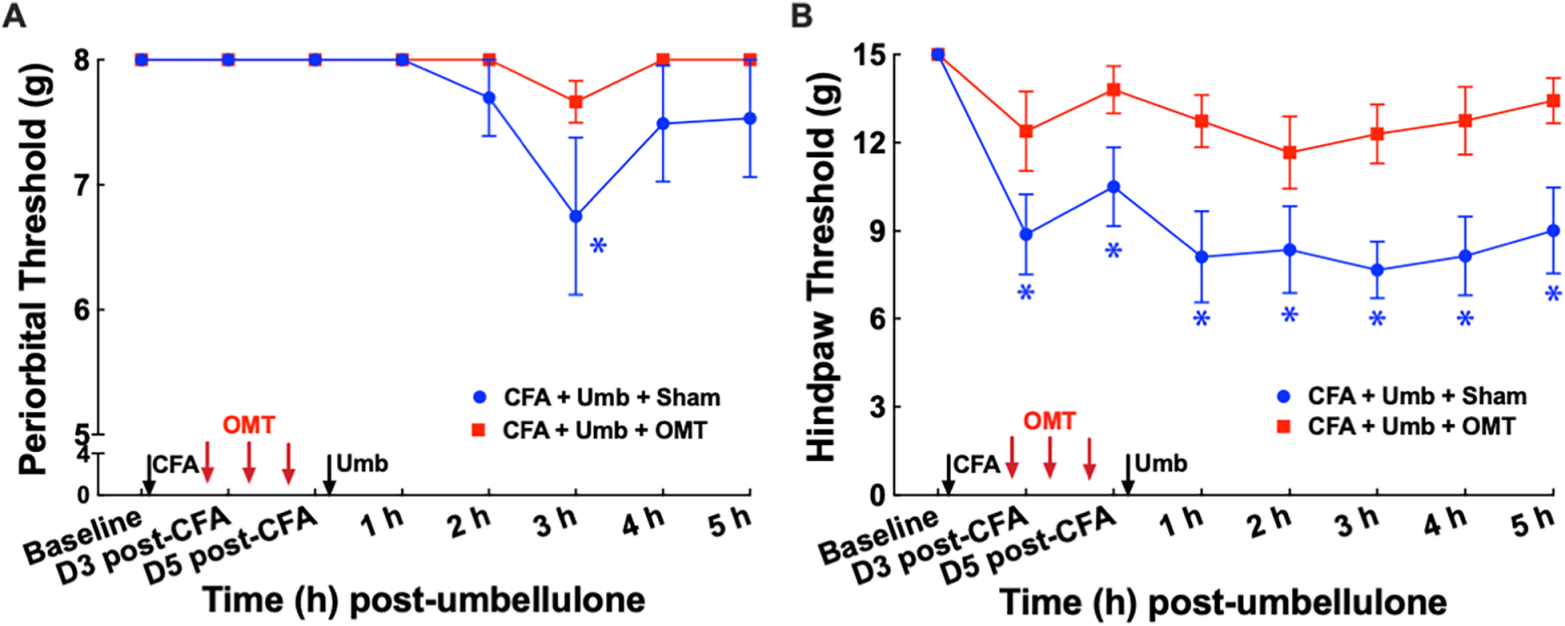
Blockade of periorbital (**A**) and hind paw (**B**) tactile allodynia by preventive OMT. Animals were treated with CFA (1:1 dilution with saline) and umbellulone (50 mM, 50 μl) or its vehicle (5% DMSO/95% H_2_O). OMT was applied on Days 1, 3 and 5 after CFA injections prior to umbellulone inhalation as a preventive treatment. Umbellulone significantly reduced periorbital and hind paw thresholds selectively in CFA-primed rats. However, application of OMT completely prevented the development of periorbital and hind paw allodynia induced by umbellulone in CFA-primed rats. Statistical analysis was performed using two-way ANOVA *post hoc* Dunnett's multiple comparison test. *P < 0.05 compared to the baseline (BL) within the same group. *N* = 8/group.

To examine the effect of OMT as a preventive treatment for migraine-like pain, we administered 3 episodes of OMT on Days 1, 3 & 5 post-CFA in animals receiving CFA and Umbellulone treatment (Figure 4). Following umbellulone inhalation, sham-treated animals developed cutaneous allodynia as expected, shown by the significant reduction of the periorbital and hind paw threshold to 6.7 ± 0.8 and 7.7 ± 0.2 g, respectively (*P* < 0.05 vs. baseline). However, animals receiving 3 preventive applications of OMT after CFA and prior to umbellulone administration exhibited a complete blockade of cutaneous allodynia development throughout the entire time course. At 3 h post-umbellulone, their periorbital and hind paw tactile threshold remained at 7.7 ± 0.2 & 12.3 ± 0.2, respectively (*P* > 0.05 compared to the baseline). This batch of animals also exhibited significant hind paw tactile hypersensitivity at Days 3 & 5 post-CFA in sham-treated animals, which was also blocked by the OMT prophylactic treatment. At Day 3 post-CFA, the average hind paw threshold was 8.9 ± 0.4 g (*P* < 0.05 vs. baseline) and 12.4 ± 0.3 g (*P* > 0.05 vs. baseline) for sham and OMT-treated animals, respectively (*N* = 8/group).

### OMT blocked the activation of TNC neurons

**Figure 5:**
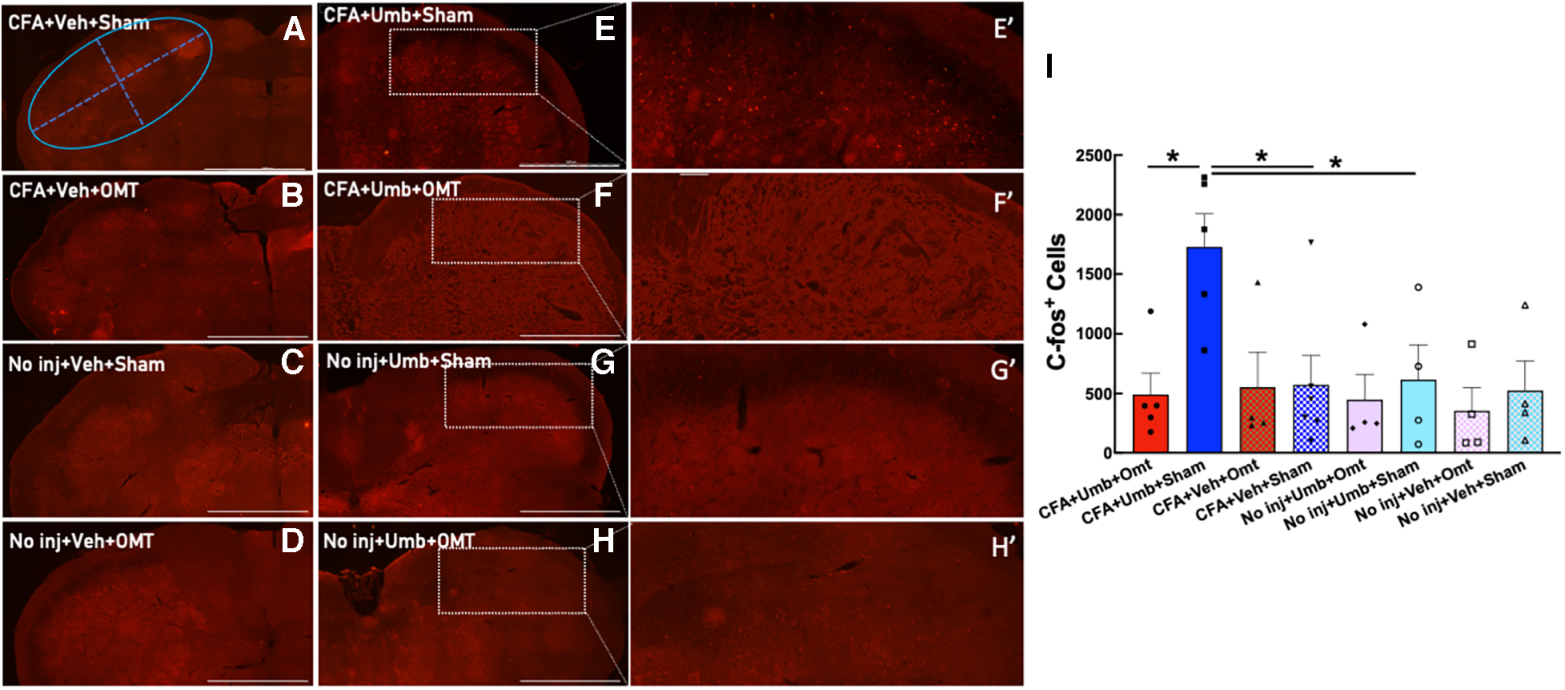
Blockade of trigeminal nucleus caudalis (TNC) activation by preventive OMT. Animals were treated with CFA (1:1 dilution with saline) and umbellulone (50 mM, 50 μl) or its vehicle (5% DMSO/95% H_2_O). OMT was applied on Days 1, 3 and 5 after CFA injections prior to umbellulone inhalation as a preventive treatment. Umbellulone significantly increased the expression of c-fos in TNC at 2 h after umbellulone treatment selectively in CFA-primed rats. However, application of OMT successfully abolished this enhancement. **E’**,**F’**,**G’**,**H’** represent amplified images of the rectangle regions in **E**,**F**,**G**,**H**. The number of c-fos positive cells in all images was quantified automatically via Cytation 5 using the same pre-set Region of Interest (ROI) illustrated by the blue oval in Panel (**A**) with the long diameter of 2,400 μm and the short diameter of 1,000 μm. The bar graph (**I**) illustrates quantified data with individual data points shown in the scatter lot. Each data point represents the average number of c-fos^+^ cells of 6 independent (non-adjacent) TNC slices from the same animal. Statistical analysis was performed using one-way ANOVA *post hoc* Tukey's multiple comparison test. *P < 0.05 compared to the corresponding group. *N* = 4–6/group. Scale bar: 1,000 μm.

To investigate the underlying mechanisms of OMT in alleviating migraine-like pain, we assessed its effects on the activation of the trigeminal system, including peripheral neurons in the TG and central neurons in the brainstem TNC. Animals received CFA and umbellulone, along with 3 applications of preventive OMT on Days 1, 3, & 5 post-CFA, as described above. At 2 h post-umbellulone inhalation, the animals were sacrificed via transcardial perfusion of the fixatives, and the TG and TNC tissues were processed immunohistochemically. We quantified the expression of c-fos, an immediate early response gene, to assess neuronal activation in the TNC. The number of c-fos^+^ cells was quantified from 6 independent slices of the TNC tissues for each animal (Figure 5). The expression of c-fos more than doubled after umbellulone inhalation in CFA-primed, sham-treated rats (CFA + Umbellulone + Sham, 1,728 ± 249) compared to the rats without priming (No injection + Umbellulone + Sham, 616 ± 253, *P* < 0.05) or primed without umbellulone inhalation (CFA + Vehicle + Sham, 574 ± 225, *P* < 0.05). However, the application of preventive OMT completely blocked this increase (CFA + Umbellulone + OMT, 492 ± 160, *P* < 0.05 vs. CFA + Umbellulone + Sham group). *N* = 4–6/group with individual group size shown in the scatter plot in Figure 5I.

### OMT suppressed the elevation of CGRP expression in TG neurons

**Figure 6:**
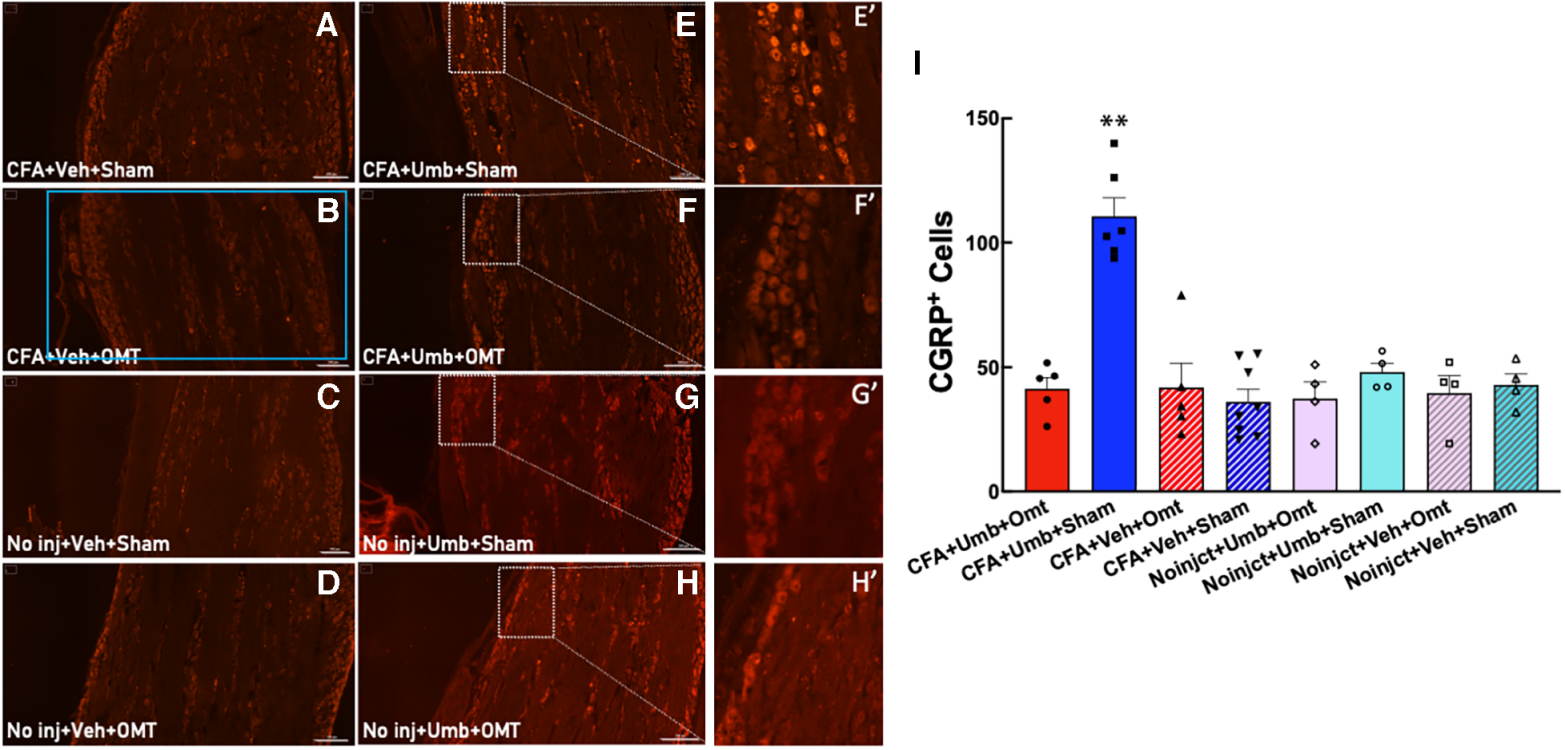
Blockade of the CGRP expression in trigeminal ganglia (TG) by preventive OMT. Animals were treated with CFA (1:1 dilution with saline) and umbellulone (50 mM, 50 μl) or its vehicle (5% DMSO/95% H_2_O). OMT was applied on Days 1, 3 and 5 after CFA injections prior to umbellulone inhalation as a preventive treatment. Umbellulone significantly increased the expression of CGRP in TG at 2 h after umbellulone treatment selectively in CFA-primed rats. However, application of OMT successfully prevented this enhancement. **E’**,**F’**,**G’**,**H’** represent amplified images of the rectangle regions in **E**,**F**,**G**,**H**. The number of CGRP positive cells in all images was quantified automatically via Cytation 5 using the same pre-set Region of Interest (ROI) illustrated by the blue rectangle in Panel (**B**) with the sides of 2,200 × 1,200 μm. The bar graph (**I**) illustrates quantified data with individual data points shown in the scatter lot. Each data point represents the average number of CGRP^+^ cells of 4 independent (non-adjacent) TG slices from the same animal. Statistical analysis was performed using one-way ANOVA *post hoc* Tukey's multiple comparison test. *P < 0.05 compared to any other group. *N* = 4–8/group. Scale bar: 200 μm.

CGRP has been identified to play a pivotal role in migraine pathophysiology. We measured CGRP expression in peripheral TG neurons at 2 h after umbellulone exposure. Animals were treated as described above, and the TG tissues were collected and processed for immunohistochemistry. The number of CGRP^+^ cells was quantified from 4 independent slices of the TG tissues for each animal (Figure 6). The results showed that CGRP expression was significantly increased in the CFA + Umbellulone + Sham group (111 ± 7) compared to the CFA-primed, but without umbellulone trigger group (CFA + Vehicle + Sham, 36 ± 5, *P* < 0.01) or the group without priming but with umbellulone trigger (No injection + Umbellulone + Sham, 48 ± 4, *P* < 0.01). However, the application of OMT on Days 1, 3 and 5 post-CFA completely blocked this increase (CFA + Umbellulone + OMT, 41 ± 4, *P* < 0.01 vs. CFA + Umbellulone + Sham group). *N* = 4–8/group with individual group size shown in the scatter plot in Figure 6I.

## Discussion

OMT presents a significant non-pharmacological strategy to treat migraine pain. However, despite its efficacy, the precise mechanisms through which OMT exerts its effects have remained unexplored, hampering its widespread utilization. In this study, we created a new rodent model to explore the impact of OMT on both behavior manifestations and physiological mechanisms underpinning migraine pathology. Our findings offer compelling evidence that multiple preventive applications of OMT in sensitized rats effectively blocked the development of cutaneous tactile allodynia—a hallmark of migraine pain. This effect appears to be mediated via the modulation of the trigeminal system, involving the inhibition of CGRP overexpression in primary sensory neurons and suppression of secondary order neuron activation within the brainstem. Interestingly, OMT applied after the umbellulone trigger partially diminished the development of allodynia, indicating that early intervention with OMT may be more effective, consistent with clinical observations (47).

Mounting evidence supports the crucial role of trigeminocervical complex activation in migraine pathophysiology. There have been multiple animal models of migraine to recapitulate different aspects of human migraine pain. The initial rat model of migraine directly activates the trigeminal neurons or the meninges by electrical stimulation or injection of inflammatory mediators (48–52). These models elicit activation of the trigeminal system to produce robust cephalic and extracephalic allodynia. However, they are invasive and produce injuries to the skull, which is absent in primary migraine headache. Subsequently, several non-invasive migraine models were created to mimic this key feature of migraine (6, 34, 53). Our novel migraine model exhibited that the TRPA1 agonist umbellulone selectively induced cephalic and extracephalic allodynia in CFA-primed animals, corroborating the observations from the Durham (34) and Porreca (35) research groups. Diverging from the Durham model that employed California bay leaf extract as the migraine trigger, we utilized pure umbellulone, as adopted by the Porreca group, to ensure precise control of the trigger dosage and circumvent inconsistencies associated with different batches of plant materials. By maintaining the umbellulone dose at a subthreshold level, we successfully avoided the induction of migraine-like pain in naïve (non-primed) animals. Additionally, we introduced mild neck inflammation through intratrapezius injections of CFA based on the Durham model to mimic the conditions often observed in patients with cervical muscle fatigue, which can trigger migraine attacks. Critically, animals receiving both CFA and umbellulone exhibited similar pathophysiological changes in the trigeminal system, including CGRP overexpression in the TG and activation of second order neurons in the TNC. Notably, these changes were effectively normalized by the prophylactic administration of OMT.

Clinically, two randomized, sham-controlled clinical trials encompassing a total of 148 chronic migraine patients have examined the therapeutic effects of OMT on migraine headaches (43, 54). Both studies reported superior efficacy of OMT over sham treatments, with multiple OMT sessions significantly reducing the frequency of migraine days per month. The treatment protocols in these trials encompassed various techniques such as myofascial release, balanced ligamentous tension, balanced membranous tension, and cranial-sacrum techniques such as suboccipital release. Drawing inspiration from these clinical maneuvers, we designed the current OMT protocol employing soft tissue and articulatory techniques to emulate clinical practice.

Our manipulations targeted the four muscles constituting the posterior suboccipital triangle: rectus capitis posterior major (RCPMa), rectus capitis posterior minor (RCPmi), obliquus capitis inferior (OCI), and obliquus capitis superior (OCS). These muscles are innervated by the dorsal ramus of C1, situated beneath the upper trapezius (innervated by spinal accessory nerve CN XI, C3 and C4) and overlay the deeper neck and throat myofascial tissues innervated by cervical spinal nerves C1–C4. It is worth noting that RCPMa and RCPmi have a connective bridge attachment to the dura mater (55, 56), establishing a potential link between tension reduction in the suboccipital triangle and headache alleviation. This tension reduction likely promotes myofascial relaxation and improved vascular flow dynamics, along with the inhibition of the first cervical nerve (C1), the greater occipital nerve (a medial branch of dorsal ramus of C2), and the lesser occipital nerve (dorsal ramus of C3). Given their heightened density of muscle spindles relative to larger muscles, OCS and RCPMa are particularly sensitive to stretch and exhibit strain with light force application (57). This phenomenon is observed across different species, similar to the calming effect experienced by human infants when caressed at the base of the skull or when baby animals are carried by the nape of their necks (58–60). Therefore, the soft tissue and articulatory techniques employed in our study may elicit relaxation in rats that is analogous to the release of muscle tension observed in humans.

It is worth noting that the OMT was performed in awake animals under brief restraint instead of anesthesia. This was based on our preliminary data that isoflurane anesthesia might interfere with the development of the cephalic pain either during the priming phase or abortive treatment phase. Thus, we chose to briefly restrain the animals to perform OMT awake. The concerns of the potential restraint stress were mitigated by the lack of tactile hypersensitivity in the CFA + Umbellulone + Sham group for both abortive and preventive experiments. Indeed, much more intense restraint stress is required to induce migraine-like pain phenotype as shown by the Porreca group. They have reported using 1 h/day × 2 days of bright light stress to trigger migraine in drug-primed rats (61) or 2 h/day × 3 days of restraint stress to prime the mice so they are sensitive to umbellulone trigger (35). The stimulus intensity of the brief 2 min restriction in our experiments was far below the migraine-eliciting ones.

The underlying pathophysiological mechanisms for OMT to alleviate cephalic pain has not been elucidated previously. Our results revealed that recurrent prophylactic OMT applications effectively inhibited CGRP overexpression in primary sensory neurons within TG and the enhanced activation of secondary order neurons in TNC prompted by umbellulone in CFA-primed rats. CGRP and its receptors are known to be expressed in TG neurons of both rodents and humans with similar patterns (62–64). CGRP is expressed in about 50% TG neurons, primarily encompassing unmyelinated small to medium-sized neurons, particularly c-fibers responsible for transmitting pain sensation (62–64). Our results demonstrated significantly higher levels of CGRP expression in TG neurons selectively in the CFA + Umbellulone + Sham group, underscoring the pivotal role of CGRP in the development of migraine in this novel model. Similarly, the augmented activation of second order neurons in TNC following the priming and migraine trigger was also consistent with other rodent migraine models and human imaging studies (65, 66). The soft tissue and articulatory techniques adopted in our study exhibited remarkable efficacy to suppress both groups of neurons, potentially representing the core molecular mechanisms driving OMT's migraine-alleviating effect.

OMT is a highly individualized treatment approach, tailored to address the specific needs of each patient. Despite the variances in individual treatment plans, similar techniques have been used to treat different kinds of headaches with underlying pathophysiological changes. For instance, myofascial release has garnered considerable success in treating tension-type headaches (67). In our study, we focused on soft tissue and articulatory techniques and their effects in a specific migraine model. Further investigations are needed to determine if these techniques can exert similar effects in other migraine models, thereby broadening our understanding of their applicability across diverse migraine types.

Overall, our research provides invaluable insights into the mechanisms underpinning the therapeutic effects of OMT for migraine-like pain. By unveiling the impact of OMT on the trigeminal system, including its ability to modulate CGRP expression and neuronal activation, we contribute substantially to the scientific understanding of OMT's physiological basis in mitigating migraines. These findings further support the clinical adoption of OMT as an efficacious non-pharmacological intervention for alleviating migraine-associated pain.

## Highlights

• Inhalation of umbellulone induced periorbital and hind paw tactile allodynia in rats sensitized with Complete Freund's Adjuvant (CFA).
• Osteopathic manipulative treatment (OMT) given as a preventive or abortive treatment blocked umbellulone-induced cutaneous allodynia.
• Preventive OMT applications curbed abnormal activation of trigeminal nucleus caudalis induced by umbellulone.
• OMT suppressed the elevation of calcitonin gene-related peptide (CGRP) expression in trigeminal ganglia induced by umbellulone inhalation.

## Data Availability

The original contributions presented in the study are included in the article/Supplementary Material, further inquiries can be directed to the corresponding author.
